# Separate roles for chromatin and lamins in nuclear mechanics

**DOI:** 10.1080/19491034.2017.1414118

**Published:** 2017-12-28

**Authors:** Andrew D. Stephens, Edward J. Banigan, John F. Marko

**Affiliations:** a Department of Molecular Biosciences, Northwestern University, Evanston, Illinois, USA; b Department of Physics and Astronomy, Northwestern University, Evanston, Illinois, USA; c Institute for Medical Engineering and Science, Massachusetts Institute of Technology, Cambridge, Massachusetts

**Keywords:** chromatin, force, lamin, micromanipulation, nucleus

## Abstract

The cell nucleus houses, protects, and arranges the genome within the cell. Therefore, nuclear mechanics and morphology are important for dictating gene regulation, and these properties are perturbed in many human diseases, such as cancers and progerias. The field of nuclear mechanics has long been dominated by studies of the nuclear lamina, the intermediate filament shell residing just beneath the nuclear membrane. However, a growing body of work shows that chromatin and chromatin-related factors within the nucleus are an essential part of the mechanical response of the cell nucleus to forces. Recently, our group demonstrated that chromatin and the lamina provide distinct mechanical contributions to nuclear mechanical response. The lamina is indeed important for robust response to large, whole-nucleus stresses, but chromatin dominates the short-extension response. These findings offer a clarifying perspective on varied nuclear mechanics measurements and observations, and they suggest several new exciting possibilities for understanding nuclear morphology, organization, and mechanics.

## Introduction

The cell nucleus is a mechanically robust and responsive structure, and its mechanics are responsible for protecting the genome and governing gene expression through both gene positioning and mechanotransduction [1]. There are two major nuclear mechanical elements - chromatin and lamins. Chromatin is the genome and its associated proteins inside and filling the nucleus. Lamins are type V intermediate filament proteins that form a polymer network shell at the nuclear periphery. Perturbing either of these mechanical components has consequences for both nuclear stability and shape. Furthermore, alterations to chromatin and the lamina occur in many human diseases [2,3]. Thus, developing both a conceptual and a quantitative understanding of nuclear mechanics is essential to both basic cell nuclear biology and studies of many human diseases.

Mechanical response of the cell nucleus has been probed by a variety of complementary experimental techniques. To interpret these various disparate measurements, it is important to identify the spatiotemporal scales probed in each experiment. At the smallest length scales, isolated *S. pombe* yeast nuclei were stretched and compressed by 50–200 nm over seconds via optical tweezers. These experiments revealed that chromatin's attachment to the nuclear periphery contributes to nuclear mechanical rigidity on these scales [4]. Another small-deformation technique used magnetic tweezers on beads attached to protein complexes (nesprin) in the nuclear envelope of mammalian nuclei to induce local deformations of 300–600 nm (≈5% strain, where strain is defined as the deformation length as a percentage of the original length of the nucleus) and observed nesprin-dependent mechanical adaptation by stiffening over tens of seconds [5]. Others have implemented micropipette aspiration to measure large, instantaneously occurring nuclear strains (150–500%) and creeping flow over hundreds of seconds on a portion of the nucleus [6–9]. These experiments suggest a major role for lamin A in determining global nuclear stiffness. Between these scales, other techniques provide important insights. Micromanipulation of nuclei within cells by a single micropipette revealed a role for vimentin and lamins in determining nuclear stability [10]. Atomic force microscopy (AFM) experiments have provided compression measurements of differently sized areas of the nucleus, and have shown that both chromatin and lamins are major contributors to nuclear mechanics [11-14].

Each of these experiments provides a unique measurement and requires a different interpretation because each one probes different mechanical attributes of the nucleus. These differences arise due to differences in area, magnitude, direction (stretch vs. compression), and duration of force application. For example, stretching a small area may only elicit a local mechanical response at the nuclear periphery whereas larger, whole-nucleus deformation involves all nuclear components. Mechanical measurements may also depend on whether force is applied to the nucleus via specific attachments (e.g., to mechanotransducing protein complexes such as nesprin [5]) or non-specific interactions (e.g., through micropipette aspiration). Furthermore, these forces can be applied to isolated nuclei or to nuclei in cells with or without an intact cytoskeleton, which can further complicate interpretation of measurements. Nonetheless, the majority of nuclear force measurements give a Young's modulus in the range 0.1 – 1 kPa and 0.1 – 1 nN/µm spring constants when they can be calculated. The diverse measurements also indicate that nuclear mechanics are controlled by multiple components. However, they do not specifically identify and measure the distinct contributions of chromatin and lamins. Recent experiments from our group address this issue.

**Figure 1.**
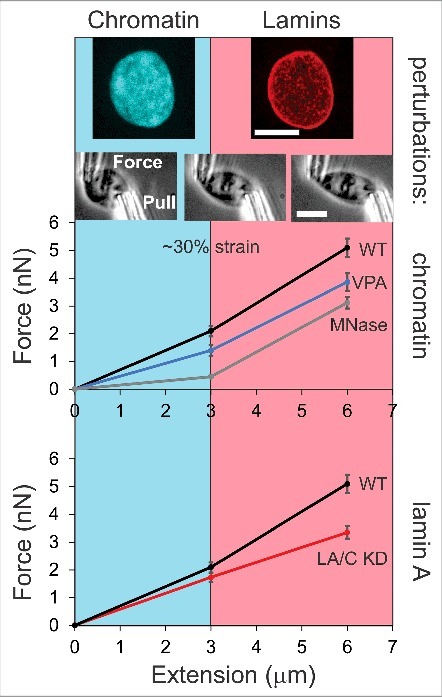
Nuclear mechanics is dictated differentially by chromatin for short extensions and lamins for long extensions through strain stiffening. Chromatin, filling the interior of the nucleus, and lamins, at the exterior, the two major mechanical components of the nucleus. In our novel micromanipulation technique we isolate a single nucleus from a living cell and attach micropipettes at each end via temporary suction and then nonspecific attachment to the pipettes. The “Pull” micropipette moves to extend the nucleus while the other “Force” micropipette's deflection provides a measure of force, due to the pipette's pre-measured spring constant (schematic of brightfield images, adapted from original article [19]). Graphs show the average force-extension data for mammalian nuclei for extensions from 0 to 3 µm (≈30% of a 10–12 µm nucleus) and from 3 to 6 µm [19], representing the chromatin-dominated short (cyan) and lamin-dominated long strain stiffening (light red) differential force regimes. The upper plot shows wild-type (WT, black line), chromatin decompaction via histone deacetylase inhibitor valproic acid (VPA, blue line), and chromatin digestion (MNase, gray line). The lower plot shows wild-type and lamin A/C knockdown (LA/C KD, red line). Error bars represent standard error. Scale bar = 10 µm.

To achieve the goal of distinguishing chromatin and lamin contributions, we developed a new method that stretches the whole nucleus at physiologically relevant strain rates of 0.01–0.1/s (15–50 nm/s) to typically observed strains of 0.1–1 (≈1–10 µm, within the range of speeds and deformations during nuclear and cell migration and other *in vivo* processes [15-17]). We modified a micromanipulation approach for isolated chromosomes [18] to isolate and stretch whole nuclei [19]. Nuclear force response was measured by attaching a micropipette to each end of the nucleus, with one “pull” micropipette moving to extend the nucleus while the other “force” micropipette remains stationary but deflects, providing a measurement of the force exerted on the nucleus (since the pipette has a pre-calibrated spring constant, see Fig. 1 images). Although we typically isolate nuclei, our experiments with nuclei remaining in cells measure a similar force response, which suggests that mechanical response of the nucleus is dominated by structural components that are stable during nuclear isolation [19].

Comparing with other studies, the measured spring constants of 0.1 – 1 nN/µm (and estimated Young's moduli of 0.2 – 0.5 kPa) in our experiments are within the range observed using other nuclear force measurement techniques (0.1 – 1 kPa, see above). Our technique produces measurements that lie at the lower end of the Young's modulus range, most likely because other force measurements are performed in the presence of the cytoskeleton, have imperfect seals and fluid flow (in the case of aspiration), and/or have high applied stresses that drive the nucleus to a strongly strain-stiffening response (as explained below). Our technique therefore presents a new, complementary measure of nuclear mechanics that, as we describe below, facilitates and provides a clarification of the relative contributions of chromatin and lamins.

## Two regimes of nuclear mechanics

A key feature of our experiments is the ability to homogeneously stretch at low and high stresses and strains across the whole nucleus. This ability provides essential information on how the nucleus responds to forces and deformations of different magnitudes. Specifically, whole-nucleus extension reveals two approximately linear force response regimes with an initial short-extension regime (Fig. 1, cyan) followed by a transition to a second, long-extension regime (Fig. 1, light red) with a 50% higher stiffness at about 30% strain (≈3 µm, Fig. 1 black line, wild-type [WT]). We refer to this force-extension behavior as strain stiffening. The consistency of the presence of these two regimes and the transition between them, as well as their distinct relative spring constants, reveals a dominant structural component for each mechanical response.

An extensive list of complementary experiments shows that chromatin dominates nuclear force response at short extensions of < 30% strain [19]. First, when perturbing chromatin alone by biochemically-driven compaction agents, isolated nuclei increased in stiffness 2–4 fold. More compellingly, experiments digesting the interior chromatin with micrococcal nuclease (MNase) resulted in a nearly complete loss of nuclear rigidity and typical force response in the short-extension regime. Although the lamins remained apparently unperturbed at the nuclear periphery, the lamina behaved as an empty, flexible polymeric meshwork shell [20] and did not provide mechanical strength until the ellipsoidal nucleus and lamina filaments collapsed along the tension axis, then providing force resistance at strains > 30% (Fig. 1 gray line, MNase). This finding reveals that lamins do not behave as a rigid structure, but instead act as a bendable, elastic polymeric network. This conclusion is also supported by recent data indicating that lamin filaments are easily bent due to their short persistence length [21,22].

Next, we demonstrated that chromatin-based rigidity is physiologically relevant by inducing histone modification state changes. Alterations to produce more euchromatin (decompact) or heterochromatin (compact) resulted in decreased or increased small-extension nuclear stiffness, respectively [19] (Fig. 1, blue line, VPA). Further research from our lab recapitulated this finding and provided new data showing that decreased heterochromatin also results in decreased small-extension nuclear stiffness [23]. Taken together, these studies provide a comprehensive cohort of data suggesting that chromatin dominates the response to short extensions (< 30%) without strongly altering the strain-stiffening behavior at longer extensions. Our data agree with other studies showing that chromatin decompaction through altered histone composition or modifications results in decreased nuclear mechanical integrity [11,24–26]. We extended these findings and provided the critical observation that chromatin dominates an entire regime of physiologically relevant strains, independent of lamins.

At a glance, these findings might seem to challenge the existing literature, but they can be reconciled by considering the effects of changing lamin A/C levels and lamin A to B stoichiometry. Through such experiments, we find that lamin A dictates the strain stiffening of the nucleus at extensions greater than 30% strains or a few µm [19]. This is in agreement with the large body of previous work that has established the importance of lamins in nuclear mechanics over the last 15 years [1-3] In particular, we find that lamin A/C knockdown, while not altering nuclear stiffness for small deformations, resulted in a loss of stiffening for extensions greater than 30% [19] (Fig. 1 red line, lamin A/C knockdown [LA/C KD]). Similarly, nuclei with naturally low levels of lamin A/C do not normally stiffen at longer extensions, but do display strain stiffening upon overexpression of lamin A. These absolute changes in nuclear strength are consistent with measurements reported by micropipette aspiration [8]. Moreover, our results highlight the significance of the fact that previous micropipette aspiration experiments typically induce strains greater than 100% and thus, likely probe the lamin-dominated long-extension mechanical regime. 

Additionally, we find that nuclei with low levels of lamin A (HEK293) are sensitive to changes in lamin A:B stoichiometry via lamin B perturbations, whereas nuclei with high lamin A content (e.g., HeLa) are not. This also echoes the prominent finding that lamin A:B stoichiometry determines nuclear and tissue mechanics [27]. Finally, our experiments observing strain stiffening provide a new explanation for the stress stiffening observed in micropipette aspiration high-strain experiments [8], where increasing aspiration pressures resulted in decreased compliance and an apparently exponential increase in Young's modulus. Altogether, our experiments show that lamins are important for robust response at large extensions through strain stiffening.

## Mechanistic model

The two-regime mechanical response of the cell nucleus can be understood through a simple quantitative simulation model in which a polymeric shell, modeling the lamina, is linked to an interior crosslinked polymeric gel, which models chromatin. This model, while conceptually simple, robustly qualitatively and quantitatively recapitulates the results of our experiments [19]. At short extensions, the stiffness of the model nucleus depends on the stiffness of the interior polymer gel that represents chromatin, while at large extensions, the stiffness of the polymeric shell that represents the lamina dominates the mechanics. As demonstrated by subsequent modeling from our group [20], this transition has a purely geometric basis. The shell behaves as a network of springs; it is initially highly flexible because the springs are not aligned with the tension axis (in the short-extension regime), but when deformed, the springs in the elongated shell are aligned with the tension, and thus strongly resist deformation (in the long-extension regime). While we do not rule out a role for nonlinear material properties of lamins, it is clear that nuclear shape alone is sufficient to produce the observed two-regime mechanical response.

Not only is the model able to recapitulate and explain features of the mechanical response of the nucleus, but it also predicts novel morphological phenomena. Specifically, the model predicts a novel buckling transition for stretched nuclear laminas lacking a stiff chromatin interior [20]. We subsequently observed this morphological failure in experiments stretching and quantitatively imaging nuclei expressing GFP-lamin A with the chromatin interior digested away via MNase treatment. However, this mechanical buckling instability is suppressed in isolated cell nuclei by the chromatin in the interior of the nucleus. Thus, the model suggests that chromatin stiffness is an important regulator of maintenance of nuclear morphology. Indeed, the latest experiments from our group [23] as well as others [11] show that the appearance of nuclear deformations called “blebs” is associated with changes in chromatin stiffness. We therefore suggest that tuning this minimalistic model and expanding it to account for chromatin's role in nuclear blebbing and other presently unexplained mechanical behaviors such as hysteresis [19] could be critical for guiding and interpreting future studies of nuclear shape and mechanics.

## Outlook: Chromatin connections

An intriguing avenue for future experimental and computational studies is in studying the complex interactions between chromatin and the lamina and the mechanical effects of internal chromatin interactions that maintain the dynamic 3-D genome [28]. Recent studies have demonstrated the importance of chromatin and its tethering to the nuclear lamina/envelope in maintaining nuclear mechanical stability [4,11,20,23], Others have shown that nuclear mechanotransduction dictating transcription requires chromatin's interaction with the lamina through proteins including HP1, BAF, SUN1/2, and emerin [29], while chromosome domains also rely on such interactions [30]. Furthermore, self-interactions within chromatin on short scales through proteins, such as HP1, have been shown to alter chromatin compaction, apparently via phase separation of heterochromatin [31,32], Other chromatin-binding factors, such as cohesin, condensin, and CTCF, bridge genomically distant regions of chromatin and could contribute to the proper transduction of force throughout the nucleus while also bolstering overall rigidity [33]. In our model, both chromatin-chromatin and chromatin-lamina linkages are essential for a robust chromatin-dominated short-extension mechanical response [19]. Since such linkages are essential factors in chromatin spatial organization, our observations suggest that chromatin organization may be involved with governing nuclear mechanical response, morphology, and mechanosensitivity.

Our finding that chromatin is a dominant mechanical component over several microns of deformation and that it can alter nuclear morphology is potentially of broad physiological significance. A number of disease phenotypes are known to exhibit abnormal nuclear morphology and mechanics, and these phenotypes have largely (although not exclusively [11]) been attributed to lamins [2]. Our work raises the intriguing possibility that chromatin mechanics and organization could play an important role in these diseases as well. For example, studies on laminopathies, such as Hutchison-Gilford progeria syndrome (HGPS), often focus on alterations to and due to lamins. However, it is now well known that many laminopathies are associated with alterations to chromatin organization [34], chromatin-associated proteins and their post-translational modifications [35], and chromatin's physical connections to the periphery [36]. Moreover, we have recently found that broad alterations to the histone modification profile can regulate nuclear blebbing and overall morphology in HGPS patient cells, as well as in other cell types [23]. We hypothesize that since lamin mutations alter interactions with a host of chromatin-binding proteins [36], perturbed chromatin mechanics and organization could contribute to disease mechanisms through altered nuclear morphology and mechanics. Thus, in future studies, it will be essential to differentiate the roles of chromatin, lamins, and their interacting proteins in these human disease scenarios.

In summary, our novel whole-nucleus extension micromanipulation experiments over a range of applied forces and resultant strains provide unique, clarifying insights into the relative mechanical contributions of chromatin and lamins. These experiments support our conceptually simple, yet quantitative and predictive simulation model of nuclear mechanics and morphology [19,20], We hope that our key finding of a chromatin-dominated short-extension regime will contribute to the growing body of work that the genome is not only a string of genetic code, but is also a major structural component that dictates cell and nuclear mechanics and nuclear morphology. Furthermore, our observation of lamin-dominated large-extension strain stiffening provides important context to years of studies that considered lamins to be the primary nuclear mechanical component. While we have detailed differentially dominated regimes, we note that both wild-type and diseased nuclei have complex alterations to both components as well as to both chromatin-chromatin and chromatin-lamina interactions. These alterations to nuclear organization likely directly dictate mechanical response of the nucleus, mechanotransduction, and gene transcription. Thus, as massive ongoing NIH efforts, such as the Physical Sciences and Oncology Network and the 4D Nucleome Project continue to unravel the complexity of nucleus and genome organization, untangling the interplay between structure/organization and mechanical behavior of the nucleus will be critical in providing further mechanistic insights into cell nuclear biology.
